# Evaluating effect of different dominance genotype encodings on genome-wide association studies and genomic selection

**DOI:** 10.5713/ab.24.0658

**Published:** 2025-03-31

**Authors:** Xiangyu Dai, Jiakun Qiao, Zhiwei Long, Zhaoxuan Che, Fangjun Xu, Na Miao, Mengjin Zhu

**Affiliations:** 1 Key Lab of Agricultural Animal Genetics, Breeding, and Reproduction of Ministry of Education, Huazhong Agricultural University, Wuhan, China; 2 The Cooperative Innovation Center for Sustainable Pig Production, Huazhong Agricultural University, Wuhan, China

**Keywords:** Dominance, Genome-wide Association Study, Genomic Selection, Genomic Prediction

## Abstract

**Objective:**

The quantification of dominance effects varies across different models, and the appropriate coding in genetic analyses remains debated. This study investigated several proposed dominance encoding methods, evaluating their performance in genetic analyses.

**Methods:**

Three datasets, each representing the breeds Duroc, Landrace, and Yorkshire, were used in this study. We assessed heritability, genetic effects, and prediction accuracy in genomic selection (GS), as well as significant loci and statistical power in genome-wide association studies (GWAS).

**Results:**

In GS, correlations among additive effects and among total genetic effects across models were high (0.9 to 1) under different dominance encodings for most traits, while only the (0, 1, 0) and (0, 2p, 4p-2) encodings maintained high correlations for all traits. The average prediction accuracy of the additive-dominance model with the (0, 1, 0) encoding increased by 2.79% and 1.69%, respectively, compared to the (0, 1, 1) and (0, 2p, 4p-2) encodings for all traits. In GWAS, the (0, 1, 0) encoding had higher statistical power compared to the (0, 1, 1) and (0, 2p, 4p-2) encodings, especially for rare variants. Additionally, different dominance encodings identified independent and distinct significant loci.

**Conclusion:**

The (0, 1, 0) encoding method generally outperforms the others in genetic analyses, while alternative encodings provide complementary insights into dominance effects. These findings provide valuable guidance for selecting dominance encodings in genetic analyses.

## INTRODUCTION

Non-additive effects refer to the effects produced by interactions between genes, including dominance and epistatic effects [1]. Dominance effects arise from interactions between alleles resulting in the effect of the heterozygous genotype not equaling to the average effect of the homozygous genotypes [2]. Originating from Mendel’s work, the dominance effect serves as a significant contributor to genetic variance, playing a pivotal role in unraveling the underlying genetic basis of complex traits [3–5].

The neglect of dominance effects in genetic evaluations and association tests has long been a prevailing issue, driven by several factors. First, the inheritance of the dominance effect is not stable, while the additive effect already accounts for the majority of phenotypic variation [6,7]. Second, while the infinitesimal model is applicable to the additive effect, its applicability to dominance effects remains to be investigated [8]. The infinitesimal model enables a robust analysis for the inheritance of quantitative traits [9,10]. Additionally, incorporating dominance effects into statistical models, particularly in the context of high-throughput big data, increases computational complexity and reduces efficiency. Nevertheless, ignoring the dominance effect in genetic analyses can lead to biased results, particularly in hybrid populations, as well as those experiencing inbreeding and outbreeding depression [11]. Empirical studies have shown that accounting for dominance effects can improve the resolution of complex traits and enhance the accuracy of genetic evaluations [12–15].

Fisher’s infinitesimal model incorporated dominance effects, laying the foundation for subsequent models [10]. Toro and Varona proposed a regression model that accounted for both additive and dominance effects [16]. Additionally, Loos advocated for the inclusion of non-additive effects in genome-wide association studies (GWAS), while Purcell et al recommended incorporating joint additive and dominance effects in association test models [17,18]. Although there is theoretical support for the inclusion of dominance effects in models, the quantification of these effects varies significantly. Zeng et al explored the advantages and potential problems of several dominance encodings [19]. Each encoding quantifies the dominance effect differently, leading to distinct partitions of genetic effects and, consequently, variations in genetic variance components and data interpretations. However, their study did not involve specific applications.

To compare the applications of different dominance encodings in greater detail, this study considered several proposed encodings. First, genotypes AA, AB, and BB were coded as 0, 1, and 0 to represent the dominance effect [1,20]. This encoding separates genetic effects into additive and dominance effects and intuitively exhibits the additional effect of heterozygous genotypes. Second, genotypes AA, AB, and BB were coded as 0, 1, and 1 to represent the dominance effect. This encoding is commonly used in association tests [7,18], but has rarely been reported in genomic selection (GS). Third, genotypes AA, AB, and BB were coded as 0, 2p, and 4p-2 to represent the dominance effect [19–22]. This encoding divides genetic effects into breeding values and dominance deviations, with the average effect of allele substitution representing the breeding values, which are crucial for studying non-additive effects as they capture most of these effects [23,24]. Furthermore, the regression model with this encoding is strictly orthogonal, ensuring that additive and dominance effects do not influence each other and exhibit a covariance of 0 between them [21,25]. Additionally, genotypes AA, AB, and BB were coded as −1/2, 1/2, and −1/2, as well as −1/3, 2/3, and −1/3 to represent the dominance effect [26,27].

We comprehensively evaluated the performance of these dominance encodings in GS and GWAS. Our analysis involved three real datasets, which included the breeds Duroc, Landrace, and Yorkshire. In GS, we compared heritability, genetic effects, and prediction accuracy under different dominance encodings. In GWAS, we assessed statistically significant loci and statistical power across different encodings. Our results provide a more detailed comparison of encodings in practical applications, serving as a reference for researchers.

## MATERIALS AND METHODS

### Ethics statement

This study was approved by the Institutional Animal Care and Use Committee of Huazhong Agricultural University (HZAUSW-2018-008).

### Data

In this study, three breeds of Duroc, Landrace, and Yorkshire were used for analyses. They shared the same traits, namely, average daily weight gain (ADG), backfat thickness (BF), and birth weight (BW). The Duroc population included 1,697 individuals and 47,257 single nucleotide polymorphisms (SNPs), the Landrace population comprised 1,280 individuals and 47,257 SNPs, and the Yorkshire population consisted of 1,578 individuals and 47,257 SNPs. The data were obtained from our laboratory. Beagle (v5.4) [28] software was used to impute missing genotype data and PLINK (v1.9) [18] software was used for quality control. SNPs with minor allele frequency < 0.01 and Hardy-Weinberg equilibrium (HWE) p-value>1e-6 were excluded from the analysis.

### Additive and dominance encodings and corresponding genomic relationship matrices

(0, 1, 0): Genotypes AA, AB, and BB were coded as 0, 1, and 2 for the additive effect, and 0, 1, and 0 for the dominance effect. Following the method proposed by Yang et al for constructing the genomic relationship matrix [29], we calculated the genomic relationship matrices for both additive and dominance effects (*G_A_* and *G_D_*_1_, respectively). The additive genomic relationship matrix remains consistent across all analyses and will not be further described.


$$\begin{array}{l} G_{A}=M_{A}M_{A}^{T}/n\;\text{and}\\ G_{D1}=M_{D1}M_{D1}^{T}/n \end{array}$$

where *M_A_*, and *M_D_*_1_ are the matrices obtained by normalizing additive and dominance genotype matrices by each column and *n* is the number of markers. For a marker *i*, *M_Ai_* and *M_D_*_1_*_i_* were calculated as


$$\begin{array}{l} M_{Ai}=\left(Z_{Ai}-2p_{i}\right)/\sqrt{2p_{i}\left(1-p_{i}\right)}\;\text{and}\\ M_{D1i}=\left(Z_{D1i}-2p_{i}\left(1-p_{i}\right)\right)/\sqrt{2p_{i}\left(1-p_{i}\right)\left(1-2p_{i}\left(1-p_{i}\right)\right)} \end{array}$$

where *Z_A_* is the additive genotype matrix, *Z_D_*_1_ is the dominance genotype matrix and *P_i_* is the frequency of allele B. Assuming HWE, we calculated the mean and variance of each marker effect in the population using the following equations.


$$\begin{array}{l} E\left(Z_{Ai}\right)=2\times p_{i}^{2}+1\times 2p_{i}\left(1-p_{i}\right)+0\times {\left(1-p_{i}\right)}^{2}=2p_{i},\\ \text{var}\left(Z_{Ai}\right)={\left(2-2p_{i}\right)}^{2}\times p_{i}^{2}+{\left(1-2p_{i}\right)}^{2}\times 2p_{i}\left(1-p_{i}\right)+{\left(0-2p_{i}\right)}^{2}\times {\left(1-p_{i}\right)}^{2}=2p_{i}\left(1-p_{i}\right),\\ E\left(Z_{D1i}\right)=0\times p_{i}^{2}+1\times 2p_{i}\left(1-p_{i}\right)+0\times {\left(1-p_{i}\right)}^{2}=2p_{i}\left(1-p_{i}\right),\\ \text{var}\left(Z_{D1i}\right)={\left(0-2p_{i}\left(1-p_{i}\right)\right)}^{2}\times p_{i}^{2}+{\left(1-2p_{i}\left(1-p_{i}\right)\right)}^{2}\times 2p_{i}\left(1-p_{i}\right)+\\ {\left(0-2p_{i}\left(1-p_{i}\right)\right)}^{2}\times {\left(1-p_{i}\right)}^{2}=2p_{i}\left(1-p_{i}\right)\left(1-2p_{i}\left(1-p_{i}\right)\right). \end{array}$$

(0, 1, 1): Genotypes AA, AB, and BB were coded as 0, 1, and 1 for the dominance effect. Using the same approach, we could get


$$G_{D2}=M_{D2}M_{D2}^{T}/n$$

where *M_D_*_2_ is the matrix obtained by normalizing the dominance genotype matrix by each column and *n* is the number of markers. For a marker *i*, *M_D_*_2_*_i_* was calculated as


$$M_{D2i}=\left(Z_{D2i}-\left(p_{i}^{2}+2p_{i}\left(1-p_{i}\right)\right)\right)/\sqrt{{\left(1-p_{i}\right)}^{2}-{\left(1-p_{i}\right)}^{4}}$$

where *Z_D_*_2_ is the dominance genotype matrix and *P_i_* is the frequency of allele B. The mean and variance were expressed as


$$\begin{array}{l} E\left(Z_{D2i}\right)=1\times p_{i}^{2}+1\times 2p_{i}\left(1-p_{i}\right)+0\times {\left(1-p_{i}\right)}^{2}=p_{i}^{2}+2p_{i}\left(1-p_{i}\right),\\ \text{var}\left(Z_{D2i}\right)={\left(1-\left(p_{i}^{2}+2p_{i}\left(1-p_{i}\right)\right)\right)}^{2}\times p_{i}^{2}+{\left(1-\left(p_{i}^{2}+2p_{i}\left(1-p_{i}\right)\right)\right)}^{2}\times \\ 2p_{i}\left(1-p_{i}\right)+{\left(0-\left(p_{i}^{2}+2p_{i}\left(1-p_{i}\right)\right)\right)}^{2}\times {\left(1-p_{i}\right)}^{2}={\left(1-p_{i}\right)}^{2}-{\left(1-p_{i}\right)}^{4}. \end{array}$$

(0, 2p, 4p-2): Genotypes AA, AB, and BB were coded as 0, 2p, and 4p-2 for the dominance effect. The genomic relationship matrix of this dominance encoding was calculated as


$$G_{D3}=M_{D3}M_{D3}^{T}/n$$

where *M_D_*_3_ is the matrix obtained by normalizing the dominance genotype matrix by each column and n is the number of markers. For a marker *i*, *M_D_*_2_*_i_* was calculated as


$$M_{D3i}=\left(Z_{D3i}-2p_{i}^{2}\right)/\sqrt{4p_{i}^{2}{\left(1-p_{i}\right)}^{2}}$$

where *Z_D_*_3_ is the dominance genotype matrix and *P_i_* is the frequency of allele B. The mean and variance were expressed as


$$\begin{array}{l} E\left(Z_{D3i}\right)=\left(4p_{i}-2\right)\times p_{i}^{2}+2p_{i}\times 2p_{i}\left(1-p_{i}\right)+0\times {\left(1-p_{i}\right)}^{2}=2p_{i}^{2},\\ \text{var}\left(Z_{D3i}\right)={\left(\left(4p_{i}-2\right)-2p_{i}^{2}\right)}^{2}\times p_{i}^{2}+{\left(2p_{i}-2p_{i}^{2}\right)}^{2}\times 2p_{i}\left(1-p_{i}\right)+\\ {\left(0-2p_{i}^{2}\right)}^{2}\times {\left(1-p_{i}\right)}^{2}=4p_{i}^{2}{\left(1-p_{i}\right)}^{2}. \end{array}$$

(−1/2, 1/2, −1/2) and (−1/3, 2/3, −1/3): Genotypes AA, AB, and BB were coded as −1/2, 1/2, and −1/2, as well as −1/3, 2/3, and −1/3 for the dominance effect. The genomic relationship matrices for these two dominance encodings were identical to those derived from the (0, 1, 0) encoding. Consequently, these two encodings were treated equivalently to the (0, 1, 0) encoding in this study.

### Estimations of genetic parameters

Two models were considered: the linear mixed model including only additive effects and the linear mixed model including additive and dominance effects.

The model including only additive effects: The linear mixed model including only additive effects is defined as


(1) $$y=\mu +g_{a}+e$$

where *y* represents the vector of phenotypic value, *μ* is the mean of the population, *g_a_* is the vector of additive effects and $g_{a}\sim N\left(0,G_{A}\sigma_{a}^{2}\right)$ where *G_A_* denotes the additive genomic relationship matrix, *e* is the vector of residual value and $e\sim N\left(0,I\sigma_{e}^{2}\right)$ where *I* is an identity matrix. The additive heritability is then expressed as


$$h_{a}^{2}=\frac{\sigma_{a}^{2}}{\sigma_{a}^{2}+\sigma_{e}^{2}}.$$

The model including additive and dominance effects: The linear mixed model including additive and dominance effects is defined as


(2) $$y=\mu +g_{a}+g_{d}+e$$

where *y* is the vector of phenotypic value, *μ* is the mean of the population, *g_a_* is the vector of additive effects and $g_{a}\sim N\left(0,G_{A}\sigma_{a}^{2}\right)$ where *G_A_* is the additive genomic relationship matrix, *g_d_* is the vector of dominance effects and $g_{d}\sim N\left(0,G_{D}\sigma_{d}^{2}\right)$ where *Gd* is the dominance genomic relationship matrix, *e* is the vector of residual value and $e\sim N\left(0,I\sigma_{e}^{2}\right)$ where *I* is an identity matrix. The additive heritability and dominance heritability are expressed as


$$\begin{array}{l} h_{a}^{2}=\frac{\sigma_{a}^{2}}{\sigma_{a}^{2}+\sigma_{d}^{2}+\sigma_{e}^{2}}\;\text{and}\\ h_{d}^{2}=\frac{\sigma_{d}^{2}}{\sigma_{a}^{2}+\sigma_{d}^{2}+\sigma_{e}^{2}}. \end{array}$$

The estimations of the above variance components were achieved using R package BGLR [30].

Furthermore, we calculated the Pearson correlation coefficients among *g_a_*, among *g_d_*, and among *g_a_*+*g_d_* across different dominance encodings.

### Genome-wide association studies

#### Statistical model

The linear mixed model in R package rMVP [31] was used to implement GWAS. For each dominance encoding, our analysis was a two-step process. The first step was to construct Equation (2) to derive the new phenotype:


$$y_{new}=y-g_{a}$$

where the additive effects were excluded. The second step was to run GWAS with the new phenotype, following the model.


(3) $$y_{new}=X_{d}b+g_{d}+e.$$

Here, *y_new_* is the reconstructed new phenotype, *b* is the effect of the test marker with the incidence *X_d_*; *g_d_* is the vector of dominance effects values of all individuals, with $g_{d}\sim N\left(0,G_{D}\sigma_{d}^{2}\right)$, where *Gd* is the dominance genomic relationship matrix; *e* is the vector of residual value, with $e\sim N\left(0,I\sigma_{e}^{2}\right)$, where *I* is an identity matrix.

#### Statistical power

According to Sham and Purcell [32], the power of GWAS can be evaluated using a chi-squared distribution with one degree of freedom (df). The formula for the non-centrality parameter (NCP) is


$$NCP=N\frac{\text{var}(X)\beta^{2}}{\sigma^{2}}$$

where *β* is the regression coefficient, *var*(*X*) is the variance of one marker under the assumption of HWE, *σ*^2^ is the residual variance, and N is the sample size. For quantitative traits, we can assume that *σ*^2^ is approximately equal to the variance of the phenotype. Then, the formula of power is


power=1-F(t,NCP,df=1)

where *F* is the cumulative distribution function of the chi-square distribution, and *t* = *F*^−1^(1−*α*, *df* = 1), with *α* representing the significance threshold and *F*^−1^ being the inverse cumulative distribution function. In this study, the remaining parameters in the NCP formula were fixed except for *var*(*X*). The parameters were set as follows: *α* = 0.05⁄*n*, *N* = 1000, *β* = 1, and the variance of the phenotype was 1. Here, *n* corresponds to the number of markers in the real data used in this study. The sample size closely reflects the real data in this study. The variance of the phenotype is equivalent to that of the normalized phenotype.

### Genomic prediction

#### The model including only additive effects

We considered a model containing both the training population *y*_1_ and the testing population *y*_2_ [33]:


$$\left[\begin{array}{l} y_{1}\\ y_{2} \end{array}\right]=\left[\begin{array}{l} \mu \\ \mu \end{array}\right]+\left[\begin{array}{l} g_{a1}\\ g_{a2} \end{array}\right]+\left[\begin{array}{l} e_{1}\\ e_{2} \end{array}\right]$$

The mean and variance of this equation are:


$$\begin{array}{l} E\left[\begin{array}{l} y_{1}\\ y_{2} \end{array}\right]=\left[\begin{array}{l} \mu \\ \mu \end{array}\right],\\ var\left[\begin{array}{l} y_{1}\\ y_{2} \end{array}\right]=\left[\begin{array}{ll} G_{A11} & G_{A12}\\ G_{A21} & G_{A22} \end{array}\right]\sigma_{a}^{2}+\left[\begin{array}{ll} I & 0\\ 0 & I \end{array}\right]\sigma_{e}^{2}. \end{array}$$

The predicted values for *y*_2_ were obtained using the following equation:


$${\hat{y}}_{2}=\mu +G_{A21}\sigma_{a}^{2}{\left(G_{A11}\sigma_{a}^{2}+I\sigma_{e}^{2}\right)}^{-1}\left(y_{1}-\mu \right)$$

The prediction accuracy was expressed as the Pearson correlation coefficient between *y*_2_ and *ŷ*_2_. To obtain a reliable estimate, we calculated the prediction accuracy 25 times using 5×5-fold cross-validation, with the mean value reported as the final result.

The model including additive and dominance effects: We also considered a model containing the training population *y*_1_ and the testing population *y*_2_:


$$\left[\begin{array}{l} y_{1}\\ y_{2} \end{array}\right]=\left[\begin{array}{l} \mu \\ \mu \end{array}\right]+\left[\begin{array}{l} g_{a1}\\ g_{a2} \end{array}\right]+\left[\begin{array}{l} g_{d1}\\ g_{d2} \end{array}\right]+\left[\begin{array}{l} e_{1}\\ e_{2} \end{array}\right]$$

The mean and variance of this equation are:


$$\begin{array}{l} E\left[\begin{array}{l} y_{1}\\ y_{2} \end{array}\right]=\left[\begin{array}{l} \mu \\ \mu \end{array}\right],\\ var\left[\begin{array}{l} y_{1}\\ y_{2} \end{array}\right]=\left[\begin{array}{ll} G_{A11} & G_{A12}\\ G_{A21} & G_{A22} \end{array}\right]\sigma_{a}^{2}+\left[\begin{array}{ll} G_{D11} & G_{D12}\\ G_{D21} & G_{D22} \end{array}\right]\sigma_{d}^{2}+\left[\begin{array}{ll} I & 0\\ 0 & I \end{array}\right]\sigma_{e}^{2}. \end{array}$$

The predicted values for *y*_2_ are:


$${\hat{y}}_{2}=\hat{\mu }+\left(G_{A21}{\hat{\sigma }}_{a}^{2}+G_{D21}{\hat{\sigma }}_{d}^{2}\right){\left(G_{A11}{\hat{\sigma }}_{a}^{2}+G_{D11}{\hat{\sigma }}_{d}^{2}+I{\hat{\sigma }}_{e}^{2}\right)}^{-1}\left(y_{1}-\mu \right)$$

The prediction accuracy was assessed in the same way as described previously.

## RESULTS

### Genetic parameters estimates under different models

We evaluated the genetic parameters of three traits across three breeds under different dominance encodings. Notable differences in heritability estimates were observed among different dominance encodings (Table 1). For additive heritability, the additive-dominance model yielded lower estimates compared to the additive-only model. The inclusion of dominance effects reduced the variance of additive effects. However, the additive-dominance model with the (0, 2p, 4p-2) encoding had the highest additive heritability, closest to the additive-only model, compared to the other encodings. This suggests that the additive effect in the additive-dominance model with the (0, 2p, 4p-2) encoding is least affected by the dominance effect when estimating additive heritability. For dominance heritability, the (0, 2p, 4p-2) encoding had the lowest estimates. These results indicate that different encodings lead to variations in the partitioning of variance components in the model. Furthermore, Table 1 reported log-likelihood values for different models. The three additive-dominance models yielded higher log-likelihood values than the additive-only model. The additive-dominance model with the (0, 1, 0) encoding yielded the highest log-likelihood values for all traits, providing the best fit to the dataset.

**Figure 1:**
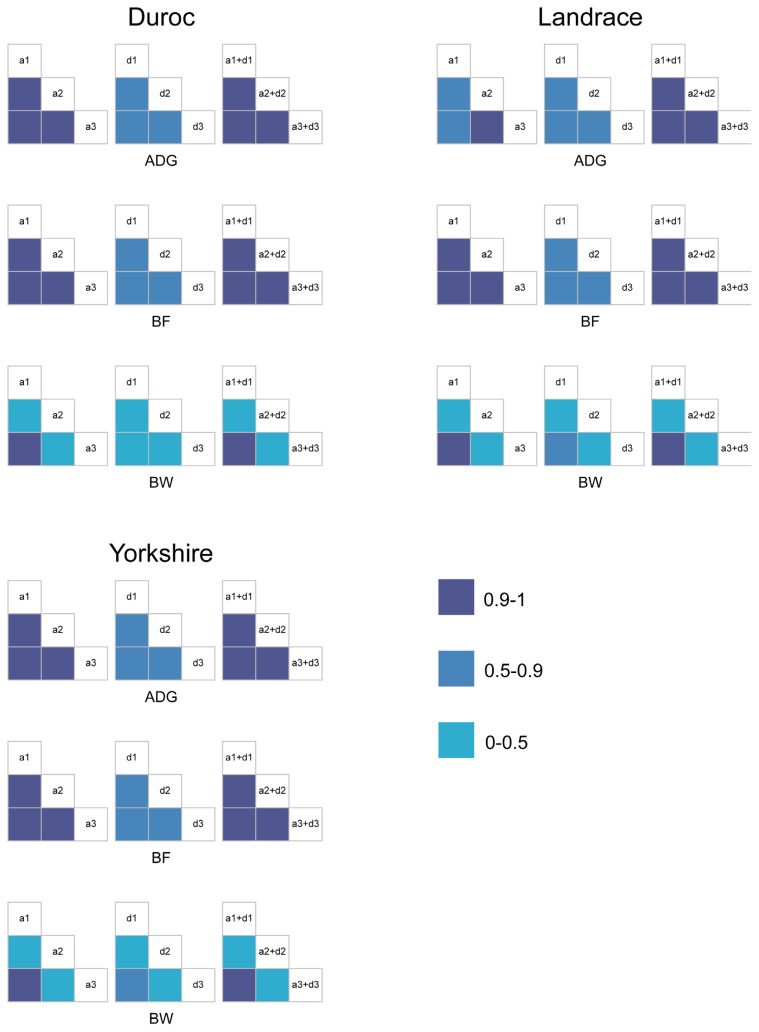
Correlations among additive effects, among dominance effects, and among total genetic effects under different models in Duroc, Landrace, and Yorkshire datasets. a1, d1, and a1+d1, additive effects, dominance effects, and total genetic effects under the model including additive and dominance effects with the (0, 1, 0) encoding; a2, d2, and a2+d2, additive effects, dominance effects, and total genetic effects under the model including additive and dominance effects with the (0, 1, 1) encoding; a3, d3, and a3+d3, additive effects, dominance effects, and total genetic effects under the model including additive and dominance effects with the (0, 2p, 4p-2) encoding; ADG, average daily weight gain; BF, backfat thickness; BW, birth weight. The correlation is divided into three gradients. 0.9-1 is high correlation, 0.5–0.9 is moderate correlation, and 0–0.5 is low correlation. Negative correlations are classified as low correlation in this study.

Different dominance encodings fit genetic effects in different ways. We looked for evidence of equivalence among encodings in the model. Figure 1 shows the correlations among genetic effects under different models, with specific values provided in Supplements 1–3. Correlations among additive effects and total genetic effects across models were high (0.9 to 1) for most traits, while only the (0, 1, 0) and (0, 2p, 4p-2) encodings maintained high correlations for all traits. In addition, correlations among dominance effects under different models were moderate (0.5 to 0.9) for most traits. These results suggest that differences in genetic effects across models primarily stem from dominance effects, and similarities exist among some encodings.

### Statistical power and dominance genome-wide association studies under different dominance encodings

**Figure 2:**
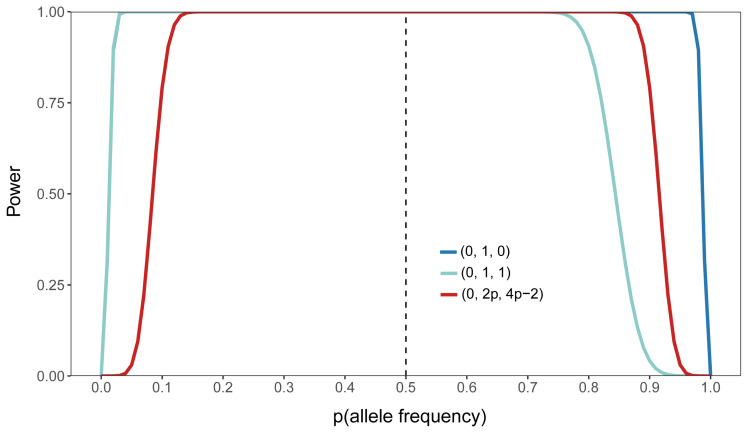
Comparison of statistical power of dominance GWAS under different dominance encodings at different allele frequencies. The vertical dashed line indicates an allele frequency of 0.5. The blue, green, and red lines represent the (0, 1, 0), (0, 1, 1), and (0, 2p, 4p-2) encodings, respectively. The curves for the (0, 1, 0) and (0, 1, 1) encodings in the left part of the figure are overlapping. GWAS, genome-wide association studies.

The models used for dominance GWAS with different dominance encodings were all linear mixed models including additive and dominance random effects. Therefore, we attempted to explore the differences in statistical power attributable to the encodings themselves. We compared the statistical power across different encodings using through a simple simulation (See Materials and Methods). The overall trends in statistical power were similar across different dominance encodings (Figure 2). As p increased, the power initially rose to a maximum and then decreased. The power of the (0, 1, 0) and (0, 1, 1) encodings was higher than that of (0, 2p, 4p-2) when p was low, while (0, 1, 0) demonstrated higher power than the other encodings at higher p values. This indicates that the (0, 1, 0) encoding has higher power for rare variants.

**Figure 3:**
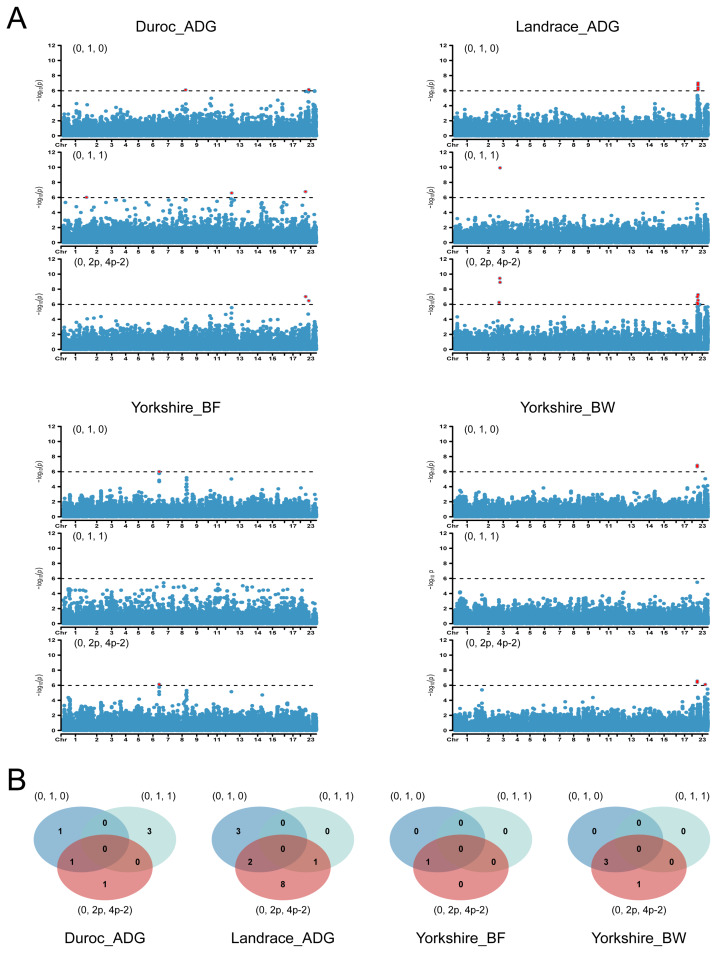
Dominance GWAS results under different dominance encodings for the four traits (Duroc_ADG, ADG in Duroc; Landrace_ADG, ADG in Landrace; Yorkshire_BF, BF in Yorkshire; Yorkshire_BW, BW in Yorkshire). (A) Manhattan plots depicting from dominance GWAS results. The horizontal dashed line indicates the significance threshold and the significant loci are highlighted in red (B) Venn diagrams illustrating the number of shared and unique significant loci. ADG, average daily weight gain; BF, backfat thickness; BW, birth weight; GWAS, genome-wide association studies.

Given the distinct properties of each dominance encoding, we employed dominance GWAS to identify specific significant loci (Figures 3A, 3B; Supplement 4). In the trait of ADG in Duroc, different dominance encodings identified independent significant loci, although one locus was commonly associated with both (0, 1, 0) and (0, 2p, 4p-2). Additionally, two loci were identified by both (0, 1, 0) and (0, 2p, 4p-2), and one locus was associated with both (0, 1, 0) and (0, 1, 1) in the trait of ADG in Landrace. Similar findings were observed in the traits of BF and BW in Yorkshire, where identical loci were associated with both (0, 1, 0) and (0, 2p, 4p-2). Notably, these loci were consistently associated with the (0, 2p, 4p-2) encoding. For other traits, different dominance encodings identified distinct significant loci. For these significant loci, we calculated the phenotypic variation explained by additive and dominance components of them separately (Supplement 5).

The results suggest that the (0, 1, 0) encoding has advantages in dominance GWAS; however, it is important to acknowledge that other encodings provide valuable, complementary information.

### Genomic prediction

**Figure 4:**
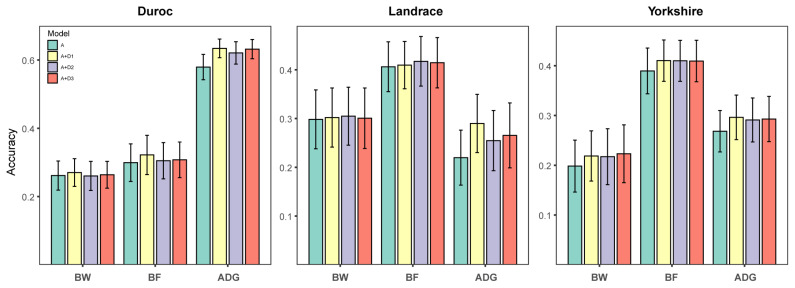
Comparison of prediction accuracy of different models in Duroc, Landrace, Yorkshire datasets. A, the model including only additive effects. A+D1, the model including additive and dominance effects with the (0, 1, 0) encoding; A+D2, the model including additive and dominance effects with the (0, 1, 1) encoding; A+D3, the model including additive and dominance effects with the (0, 2p, 4p-2) encoding; BW, birth weight; BF, backfat thickness; ADG, average daily weight gain.

The prediction accuracy of the model incorporating only additive effects, as well as the three models including additive and dominance effects, is presented in Figure 4. The prediction accuracy of the models improved considerably when the dominance effect was considered. Specifically, the average prediction accuracy of the additive-dominance model with the dominance encodings of (0, 1, 0), (0, 1, 1), and (0, 2p, 4p-2) increased by 8.99%, 5.85%, and 7.01%, respectively, across all traits. Furthermore, the model with the (0, 1, 0) encoding demonstrated superior accuracy, with an increase of 2.79% and 1.69% compared to the models using the (0, 1, 1) and (0, 2p, 4p-2) encodings, respectively. Additionally, the (0, 2p, 4p-2) encoding outperformed the (0, 1, 1) encoding by 1.04%. These results indicate that considering dominance effects in the model improves prediction accuracy, with the (0, 1, 0) encoding providing the most accurate genomic predictions, while the (0, 1, 1) encoding being the least effective.

## DISCUSSION

A comprehensive comparison of different dominance encodings in GS and GWAS revealed both differences and similarities. Differences were evident in heritability, prediction accuracy, statistical power and significant loci across different dominance encodings, while similarities manifested in the correlations among genetic effects and significant loci. This comparison aimed to identify the dominance encoding that performs best in genetic analyses and to uncover additional insights provided by different encodings

The inclusion of dominance effects in the model led to a reduction in the variance of additive effects, especially for the (0, 1, 0) and (0, 1, 1) encodings. As noted by Su et al [1], the inclusion of dominance effects reduces the contribution of additive effects. The correlation between additive and dominance genotype matrices in the model affects the estimation of the additive variance. Zhu et al [21] calculated the covariance between additive and dominance genotype matrices in the HWE population as 2p(1-p)(1-2p) for the (0, 1, 0) encoding whereas we derived 2p(1-p)^2^ for the (0, 1, 1) encoding. Furthermore, the additive heritability in the additive-dominance model with the (0, 2p, 4p-2) encoding was close to the additive heritability in the additive-only model, aligning with the findings of Vitezica et al [20]. The covariance between additive and dominance genotype matrices with the (0, 2p, 4p-2) encoding was calculated to be 0, indicating that the model is strictly orthogonal. This model treats additive effects as breeding values, which is consistent with the additive-only model in the study by Vitezica et al [20]. However, in the additive-only model, there was no distinction between the additive effect and the average effect of allele substitution due to the exclusion of dominance effects; this distinction is not a critical factor in this context. The differing covariances between additive and dominance genotype matrices across various encodings led to differences in the partitioning and interpretation of genetic variance components.

Genetic effects can be effectively represented by parameters in different models. For the additive-dominance model with the (0, 1, 0) encoding, the values of the total genetic effects for genotypes AA, AB, and BB are 0a+0d, 1a+1d, and 2a+0d. For the (0, 1, 1) encoding, these values are 0a+0d, 1a+1d, and 2a+1d, while for the (0, 2p, 4p-2) encoding, they are 0(a+(1-2p)d)+0d, 1(a+(1-2p)d)+2pd, and 2(a+(1-2p)d)+(4p-2)d. These values clearly demonstrate the partitioning of additive and dominance effects. While the additive effect values maintain high correlations due to consistent quantification, the dominance effect values exhibit distinct features for each encoding. This is the essential difference among the encodings. Furthermore, the total genetic effect values for the (0, 1, 0) and (0, 2p, 4p-2) encodings are identical; they simply divide the genetic effects in different ways.

A simple simulation study showed the advantage of the (0, 1, 0) encoding in terms of statistical power. The variance of markers under different dominance encodings, determined by allele frequency, is described in the Materials and Methods section. The power results reflected the trend of marker variance when other parameters were fixed in the power formula. For rare variants (p<0.1 or p>0.9), the (0, 1, 1) and (0, 2p, 4p-2) encodings performed poorly. Sham and Purcell [32] noted that this power formula is less effective for rare variants in additive GWAS, and this issue is more pronounced in dominance GWAS. First, dominance effects have a smaller effect on phenotype compared to additive effects. Second, the variance of markers under dominance encodings is lower than that under the additive encoding for the same markers because there are more terms that are greater than 0 and less than 1 in the variance formula for dominance encodings.

Dominance effects can be divided into incomplete dominance, complete dominance, and overdominance depending on the degree of dominance [34,35]. Different dominance encodings have different sensitivities to the degree of dominance due to differences in quantification methods. The (0, 1, 0) and (0, 1, 1) encodings are specifically designed to capture overdominance and dominance effects, respectively [7]. In contrast, the (0, 2p, 4p-2) encoding demonstrates greater flexibility, as its values vary depending on allele frequency. For instance, when p = 0.5, the (0, 2p, 4p-2) encoding yields values identical to those of the (0, 1, 0) encoding, and as p approaches 1, it closely resembles the (0, 1, 1) encoding.

Considering dominance effects in models improved the predictive ability of models, consistent with findings from previous studies [14,36,37]. Among the evaluated dominance encodings, the (0, 1, 0) encoding outperformed the others. This encoding highlights the additional effects of heterozygous genotypes, thereby capturing dominance effects of markers more effectively. In contrast, the (0, 1, 1) encoding may have limited capacity to capture dominance effects, as it also reflects the effect of one homozygous genotype. The (0, 2p, 4p-2) encoding introduces uncertainty due to its dependence on allele frequency.

In summary, we have discussed the characteristics and potential limitations of different dominance encodings. The (0, 1, 0) encoding offers clear advantages in terms of statistical power and genomic prediction. However, the (0, 1, 1) encoding seems to be inappropriate for GS because it includes additive effects, leading to an incorrect partitioning of genetic effects. The (0, 2p, 4p-2) encoding shows promise in estimating variance components, although further investigation is needed for confirmation. We encourage researchers to consider different dominance encodings when conducting dominance GWAS.

While our study provides valuable insights into the strengths and limitations of various dominance encodings, it is important to acknowledge that the assumption of equal additive and dominance effects across quantitative trait loci (QTLs) may not always hold. Dominance effects often exhibit greater variation among QTLs compared to additive effects, potentially leading to biased construction of the dominance genomic relationship matrix. Liu et al [38] addressed this issue by weighting loci according to their degree of dominance, aligning the model assumptions with the true distribution of dominance effects. Given that different dominance encodings provide different information, future research could benefit from integrating these encodings to enhance the accuracy and interpretability of genetic analyses.

## CONCLUSION

In conclusion, this study revealed differences and similarities among different dominance encodings. The encodings exhibit varying sensitivities to the degree of dominance, providing diverse insights into genetic effects. Meanwhile, the high correlations among genetic effects across encodings suggest similar dominance mechanisms. The (0, 1, 0) encoding demonstrates superior performance in genetic analyses, while other encodings provide valuable information for resolving dominance effects.

## Figures and Tables

**Table 1:** Additive and dominance heritabilities estimates (standard errors) under different models in Duroc, Landrace, and Yorkshire datasets

Breed	Trait	Model	$h_{\text{a}}^{2}$	$h_{\text{a}}^{2}$	Log-likelihood value
Duroc	ADG	A	0.4785(0.0063)	-	2,842.9876
		A+D1	0.2619(0.0091)	0.4060(0.0116)	3,204.2851
		A+D2	0.1567(0.0257)	0.4609(0.0361)	3,126.6974
		A+D3	0.3749(0.0079)	0.2449(0.0123)	3,176.5004
	BF	A	0.2786(0.0084)	-	−3,336.0855
		A+D1	0.1845(0.0112)	0.2380(0.0199)	−3,193.8790
		A+D2	0.2247(0.0199)	0.0673(0.0226)	−3,324.3307
		A+D3	0.2584(0.0010)	0.0332(0.0093)	−3,316.3957
	BW	A	0.2735(0.0102)	-	−166.5982
		A+D1	0.1574(0.0148)	0.2057(0.0190)	−37.1528
		A+D2	0.1611(0.0130)	0.4589(0.0106)	−166.6361
		A+D3	0.2325(0.0117)	0.0129(0.0087)	−156.4410
Landrace	ADG	A	0.2302(0.0086)	-	1,921.2663
		A+D1	0.1176(0.0081)	0.2941(0.0150)	2,063.5379
		A+D2	0.1368(0.0107)	0.0934(0.0114)	1,951.6873
		A+D3	0.1907(0.0100)	0.0771(0.0073)	1,964.7485
	BF	A	0.3717(0.0075)	-	−2,429.1100
		A+D1	0.2757(0.0093)	0.1808(0.0114)	−2,357.6555
		A+D2	0.2663(0.0153)	0.0879(0.0129)	−2,409.5061
		A+D3	0.3117(0.0083)	0.0498(0.0040)	−2,403.6985
	BW	A	0.2436(0.0081)	-	−158.5726
		A+D1	0.1784(0.0103)	0.1645(0.0162)	−73.1102
		A+D2	0.1497(0.0105)	0.1078(0.0084)	−132.7963
		A+D3	0.2124(0.0093)	0.0353(0.0041)	−140.0023
Yorkshire	ADG	A	0.2583(0.0099)	-	2,640.7769
		A+D1	0.1531(0.0106)	0.2066(0.0135)	2,721.9229
		A+D2	0.1624(0.0150)	0.1620(0.0229)	2,682.6477
		A+D3	0.2339(0.0101)	0.0467(0.0073)	2,674.8039
	BF	A	0.3437(0.0074)	-	−3,019.3317
		A+D1	0.2613(0.0091)	0.1996(0.0141)	−2,904.4788
		A+D2	0.1689(0.0202)	0.2273(0.0292)	−2,951.1975
		A+D3	0.3129(0.0073)	0.0597(0.0095)	−2,962.0638
	BW	A	0.2136(0.0082)	-	2.3709
		A+D1	0.1391(0.0082)	0.1578(0.0110)	81.0912
		A+D2	0.1956(0.0090)	0.0882(0.0096)	17.4934
		A+D3	0.1865(0.0084)	0.0382(0.0044)	25.8748

A, the model including only additive effects; A+D1, the model including additive and dominance effects with the (0, 1, 0) encoding; A+D2, the model including additive and dominance effects with the (0, 1, 1) encoding; A+D3, the model including additive and dominance effects with the (0, 2p, 4p-2) encoding; ADG, average daily weight gain; BF, backfat thickness; BW, birth weight.

## SUPPLEMENTARY MATERIAL

**Supplement 1**. Correlations among additive effects under different models in Duroc, Landrace, and Yorkshire datasets.

**Supplement 2**. Correlations among dominance effects under different models in Duroc, Landrace, and Yorkshire datasets.

**Supplement 3**. Correlations among total genetic effects under different models in Duroc, Landrace, and Yorkshire datasets.

**Supplement 4**. Dominance GWAS results under different dominance encodings for the five traits (Duroc_BF, BF in Duroc; Duroc_BW, BW in Duroc; Landrace_BF, BF in Landrace; Landrace_BW, BW in Landrace; Yorkshire_ADG, ADG in Yorkshire).

**Supplement 5**. Phenotypic variation explained by additive and dominance components of significant SNPs under different dominance encodings in Duroc, Landrace and Yorkshire datasets.
