# Supplementary material for: Evaluating effect of different dominance genotype encodings on genome-wide association studies and genomic selection
Source: Anim Biosci. 2025 Mar 31;38(10):2067–78. doi: 10.5713/ab.24.0658 (PMC12415359; doi:10.5713/ab.24.0658)
Supplement: Supplementary file 1 [file ab-24-0658-Supplementary-1.pdf]

Supplement 1. Correlations among additive effects under different models in Duroc, Landrace, and Yorkshire datasets

Breed	Trait	a1-a2	a1-a3	a2-a3
Duroc	ADG	0.9461	0.9745	0.9764
	BF	0.9619	0.9638	0.9787
	BW	-0.2453	0.9642	-0.1998
Landrace	ADG	0.8522	0.8877	0.9783
	BF	0.9715	0.9779	0.9924
	BW	0.1156	0.9790	0.1486
Yorkshire	ADG	0.9319	0.9501	0.9752
	BF	0.9329	0.9723	0.9734
	BW	0.2572	0.9547	0.2972

a1-a2, the correlation between additive effects in the model including additive and dominance effects with the (0, 1, 0) encoding and the model with the (0, 1, 1) encoding. a1-a3, the correlation between additive effects in the model including additive and dominance effects with the (0, 1, 0) encoding and the model with the (0, 2p, 4p-2) encoding. a2-a3, the correlation between additive effects in the model including additive and dominance effects with the (0, 1, 1) encoding and the model with the (0, 2p, 4p-2) encoding. ADG, average daily weight gain; BF, backfat thickness; BW, birth weight.
