# Supplementary material for: Evaluating effect of different dominance genotype encodings on genome-wide association studies and genomic selection
Source: Anim Biosci. 2025 Mar 31;38(10):2067–78. doi: 10.5713/ab.24.0658 (PMC12415359; doi:10.5713/ab.24.0658)
Supplement: Supplementary file 2 [file ab-24-0658-Supplementary-2.pdf]

Supplement 2. Correlations among dominance effects under different models in Duroc, Landrace, and Yorkshire datasets

Breed	Trait	d1-d2	d1-d3	d2-d3
Duroc	ADG	0.8674	0.8890	0.7384
	BF	0.7193	0.5548	0.7048
	BW	0.1281	0.3718	0.0591
Landrace	ADG	0.7758	0.7665	0.8769
	BF	0.6913	0.6503	0.8991
	BW	0.2349	0.6636	0.1727
Yorkshire	ADG	0.8236	0.6241	0.6706
	BF	0.7524	0.6450	0.6979
	BW	0.2042	0.5581	0.1170

d1-d2, the correlation between dominance effects in the model including additive and dominance effects with the (0, 1, 0) encoding and the model with the (0, 1, 1) encoding. d1-d3, the correlation between dominance effects in the model including additive and dominance effects with the (0, 1, 0) encoding and the model with the (0, 2p, 4p-2) encoding. d2-d3, the correlation between dominance effects in the model including additive and dominance effects with the (0, 1, 1) encoding and the model with the (0, 2p, 4p-2) encoding. ADG, average daily weight gain; BF, backfat thickness; BW, birth weight.
