# Supplementary material for: Evaluating effect of different dominance genotype encodings on genome-wide association studies and genomic selection
Source: Anim Biosci. 2025 Mar 31;38(10):2067–78. doi: 10.5713/ab.24.0658 (PMC12415359; doi:10.5713/ab.24.0658)
Supplement: Supplementary file 3 [file ab-24-0658-Supplementary-3.pdf]

Supplement 3. Correlations among total genetic effects under different models in Duroc, Landrace, and Yorkshire datasets

Breed	Trait	a1+d1-a2+d2	a1+d1-a3+d3	a2+d2-a3+d3
Duroc	ADG	0.9921	0.9948	0.9964
	BF	0.9567	0.9614	0.9928
	BW	-0.0219	0.9538	-0.0647
Landrace	ADG	0.9323	0.9559	0.9830
	BF	0.9789	0.9839	0.9946
	BW	0.1678	0.9753	0.1492
Yorkshire	ADG	0.9711	0.9664	0.9858
	BF	0.9831	0.9810	0.9927
	BW	0.2578	0.9535	0.2579

a1+d1-a2+d2, the correlation between total genetic effects in the model including additive and dominance effects with the (0, 1, 0) encoding and the model with the (0, 1, 1) encoding. a1+d1-a3+d3, the correlation between total genetic effects in the model including additive and dominance effects with the (0, 1, 0) encoding and the model with the (0, 2p, 4p-2) encoding. a2+d2-a3+d3, the correlation between total genetic effects in the model including additive and dominance effects with the (0, 1, 1) encoding and the model with the (0, 2p, 4p-2) encoding. ADG, average daily weight gain; BF, backfat thickness; BW, birth weight.
