# Supplementary material for: Evaluating effect of different dominance genotype encodings on genome-wide association studies and genomic selection
Source: Anim Biosci. 2025 Mar 31;38(10):2067–78. doi: 10.5713/ab.24.0658 (PMC12415359; doi:10.5713/ab.24.0658)
Supplement: Supplementary file 4 [file ab-24-0658-Supplementary-4.pdf]

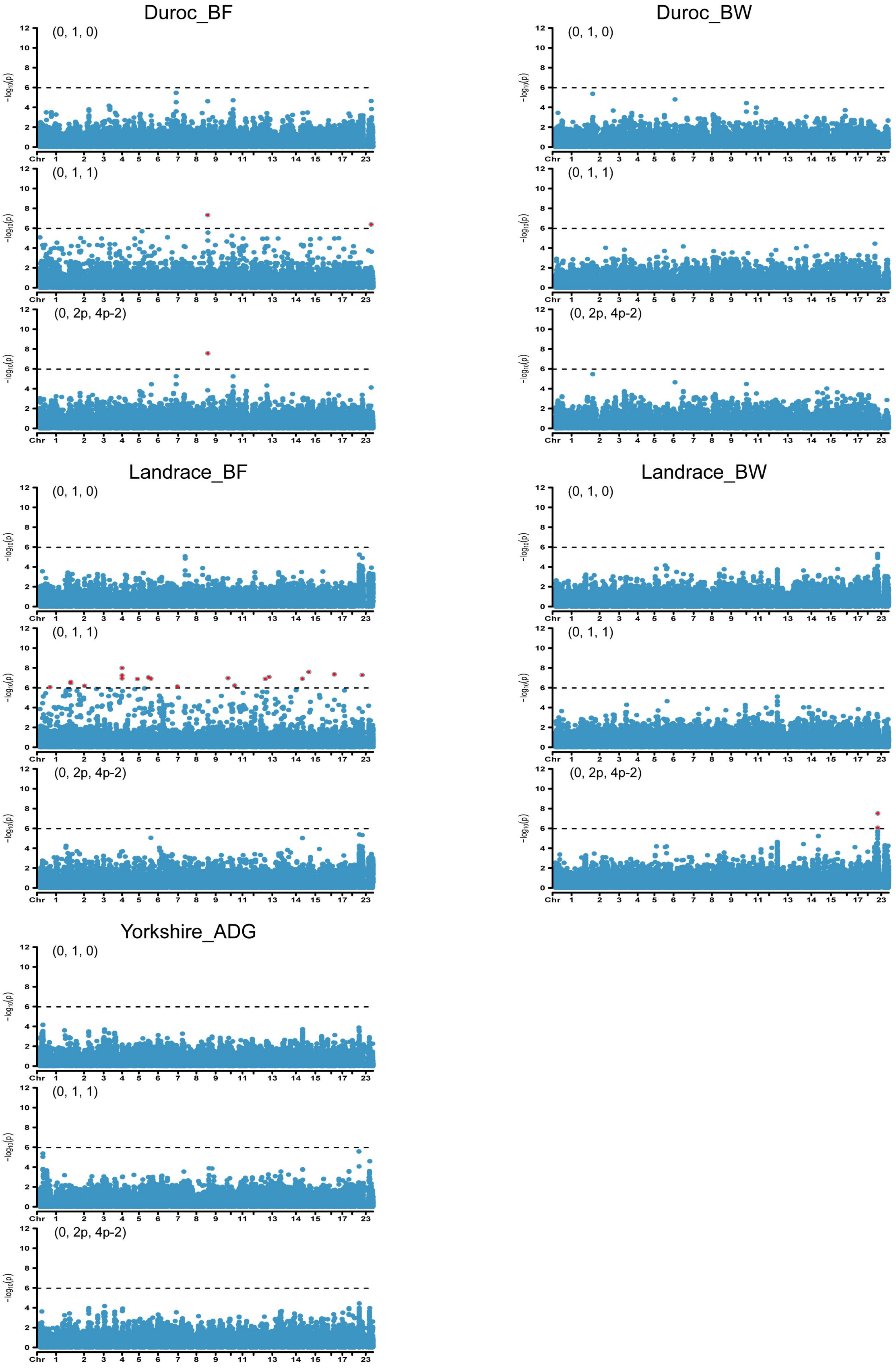

Supplement 4. Dominance GWAS results under different dominance encodings for the five traits (Duroc_BF, BF in Duroc; Duroc_BW, BW in Duroc; Landrace_BF, BF

in Landrace; Landrace_BW, BW in Landrace; Yorkshire_ADG, ADG in Yorkshire). Manhattan plots depicting from dominance GWAS results. The horizontal dashed line indicates the significance threshold and the significant loci are highlighted in red. ADG, average daily weight gain; BF, backfat thickness; BW, birth weight.
