# Supplementary material for: Evaluating effect of different dominance genotype encodings on genome-wide association studies and genomic selection
Source: Anim Biosci. 2025 Mar 31;38(10):2067–78. doi: 10.5713/ab.24.0658 (PMC12415359; doi:10.5713/ab.24.0658)
Supplement: Supplementary file 5 [file ab-24-0658-Supplementary-5.pdf]

Supplement 5. Phenotypic variation explained by additive and dominance components of significant SNPs under different dominance encodings in Duroc, Landrace and Yorkshire datasets

Breeds	Trait	Dominance encoding	PVE (additive component) (%)	PVE (dominance component) (%)
Duroc	ADG	(0, 1, 0)	1.54	5.51
		(0, 1, 1)	0.76	6.85
		(0, 2p, 4p-2)	0.40	0.23
	BF	(0, 1, 1)	0.09	1.77
		(0, 2p, 4p-2)	24.52	18.74
Landrace	ADG	(0, 1, 0)	0.10	1.26
		(0, 1, 1)	17.20	49.40
		(0, 2p, 4p-2)	0.29	6.32
	BF	(0, 1, 1)	1.17	0.63
		(0, 2p, 4p-2)	0.06	1.17
Yorkshire	BF	(0, 1, 0)	21.05	25.79
		(0, 2p, 4p-2)	26.39	22.73
	BW	(0, 1, 0)	0.12	0.23
		(0, 2p, 4p-2)	0.26	0.47

PVE (additive component), phenotypic variation explained by the additive component of significant SNPs. PVE (dominance component), phenotypic variation explained by the dominance component of significant SNPs. The significant SNPs for each trait,

identified by different dominance encodings, are based on the dominance GWAS results of this study. ADG, average daily weight gain; BF, backfat thickness; BW, birth weight.
